# Biodiversity and biological applications of marine actinomycetes—Abu-Qir Bay, Mediterranean Sea, Egypt

**DOI:** 10.1186/s43141-023-00612-8

**Published:** 2023-11-28

**Authors:** Ghada E. Hegazy, Zakia A. Olama, G. M. Abou-Elela, Heba S. Ramadan, Walaa M. Ibrahim, Dalia El S. El Badan

**Affiliations:** 1 National Institute of Oceanography and Fisheries (NIOF), Qaitbay Sq, El-Anfousy, Alexandria, 11865 Egypt; 2 Botany & Microbiology Department, Faculty of Science, Alexandria University, Alexandria, Egypt; 3 Department of Biological Sciences, Faculty of Science, Beirut Arab University, Beirut, Lebanon

**Keywords:** Natural pigments, Marine actinomycetes biodiversity, Anti-inflammatory

## Abstract

**Background:**

The ability of actinomycetes to produce bioactive secondary metabolites makes them one of the most important prokaryotes. Marine actinomycetes are one of the most important secondary metabolites producers used for pharmaceuticals and other different industries.

**Results:**

In this study, the promising actinomycetes were isolated from Abu-Qir Bay. Four different media named as starch nitrate, starch casein, glycerol asparagine, and glycerol glycine were used as a preliminary experimental media to study the role of the medium components on the counts of actinomycetes in sediment samples. The results indicated that starch casein medium reported the highest counts (30–63 CFU/g) in all the tested sites. Lower counts were detected on starch nitrate and glycerol asparagine. On the other hand, glycerol glycine medium gave the lowest counts (15–48 CFU/g). Abu-Qir_8_ harbored the highest average count of actinomycetes (63 CFU/g), followed by Abu-Qir_1_ (48 CFU/g). The lower counts were detected in Abu-Qir_5_ and Abu-Qir_7_ (26 and 29 CFU/g, respectively). A total of 12 pure obtained actinomycetes isolates were subjected to morphological, physiological, and biochemical characterization. The selected actinobacterial isolates were subjected to numerical analysis, and the majority of isolates were grouped into four main clusters (A, B, C, & D), and each of them harbored two isolates; additionally, four isolates did not cluster at this similarity level. Isolate W4 was carefully chosen as the most promising pigment and antimicrobial agent’s producer; the produced pigment was extracted and optimized by statistical experiments (PBD & BBD) and was tested for its anti-inflammatory activity. The results showed anti-inflammatory effect and prevented the denaturation of BSA protein at a concentration much higher than the safe dose and increased with increasing the pigment concentration.

**Conclusion:**

Marine actinomycetes play a vital role in the production of novel and important economic metabolites that have many industrial and pharmaceuticals applications. *Streptomyces* genera are the most important actinomycetes that produce important metabolites as previously reported.

**Supplementary Information:**

The online version contains supplementary material available at 10.1186/s43141-023-00612-8.

## Background

Actinomycetes are generally found in man-made and natural habitats; they are capable of living in different natural environments. Actinomycetes are gram +ve bacteria and are characterized by the ability to form the substrate and aerial mycelium when grown on solid media; also, they can form spores with different surfaces and have DNA with high cytosine + guanine content [1]. The actinomycetes distribution in aquatic habitats are still undiscovered, and their presence in oceans is a mystery. This may be due to the attempted discovery in detection of marine actinomycetes genera. Actinomycetes live in unusual environments, for example, in gas hydrate reservoirs of marine deep sea and aggregates of organic matter, where they are considered as the main nutrients of the microbial communities [2]. Marine actinomycetes are considered very important marine microorganisms due to their vital role in biotechnological and biological applications because they account for more than 50% of all bioactive secondary metabolites known in nature [3]. According to chemical and morphological criteria, they have been categorized into distinct genera and represent a very important role in the degradation of organic matter. Actinomycetes belong to distinct genera like *Corynebacterium*, *Actinomyces*, *Frankia*, *Arthrobacter*, *Micrococcus*, *Micromonospora*, and *Streptomyces* which are capable of producing different compounds with wide biological potentials. *Streptomyces* is the most common actinomycetes genera that were isolated. Also, *Streptomyces* are considered as a rich source of biologically active secondary metabolites [4]. The pigments are produced as secondary metabolites from different types of *Streptomyces*, and considered as one of the most interesting pigments which produced genera, this due to its high activity of production, and also because one of the most important produced pigment used in industry, melanin, can be produced by this type of bacterium. Also, this bacterium has large genetically distribution which is used easily for replication in the industry and biotechnological applications. The benefits of these natural pigments include fast and easy growth in economic uses, independence from different colors, shades, and climatic conditions. These microbial pigments production is considered as one of the most promising research fields, revealing its importance in many and different industrial and medical applications [5]. Much research has been done to isolate pigmented actinomycetes and screen them for their biological activities, for example, some microbial pigments have been reported for their anticancer activity, antiviral activity, anti-inflammatory capacity, and anti-oxidant activity and characterized by their stability to heat, light, and pH and used in food, cosmetics, textiles, and pharmaceuticals applications [6]. There are many novel actinomycetes strains isolated from marine sediment samples from the Mediterranean Sea, in Egypt. The pigment is extracted, purified, and characterized from the most promising actinomycetes isolate and then tested for its biological activities.

## Methods

### Sampling sites

**Fig. 1:**
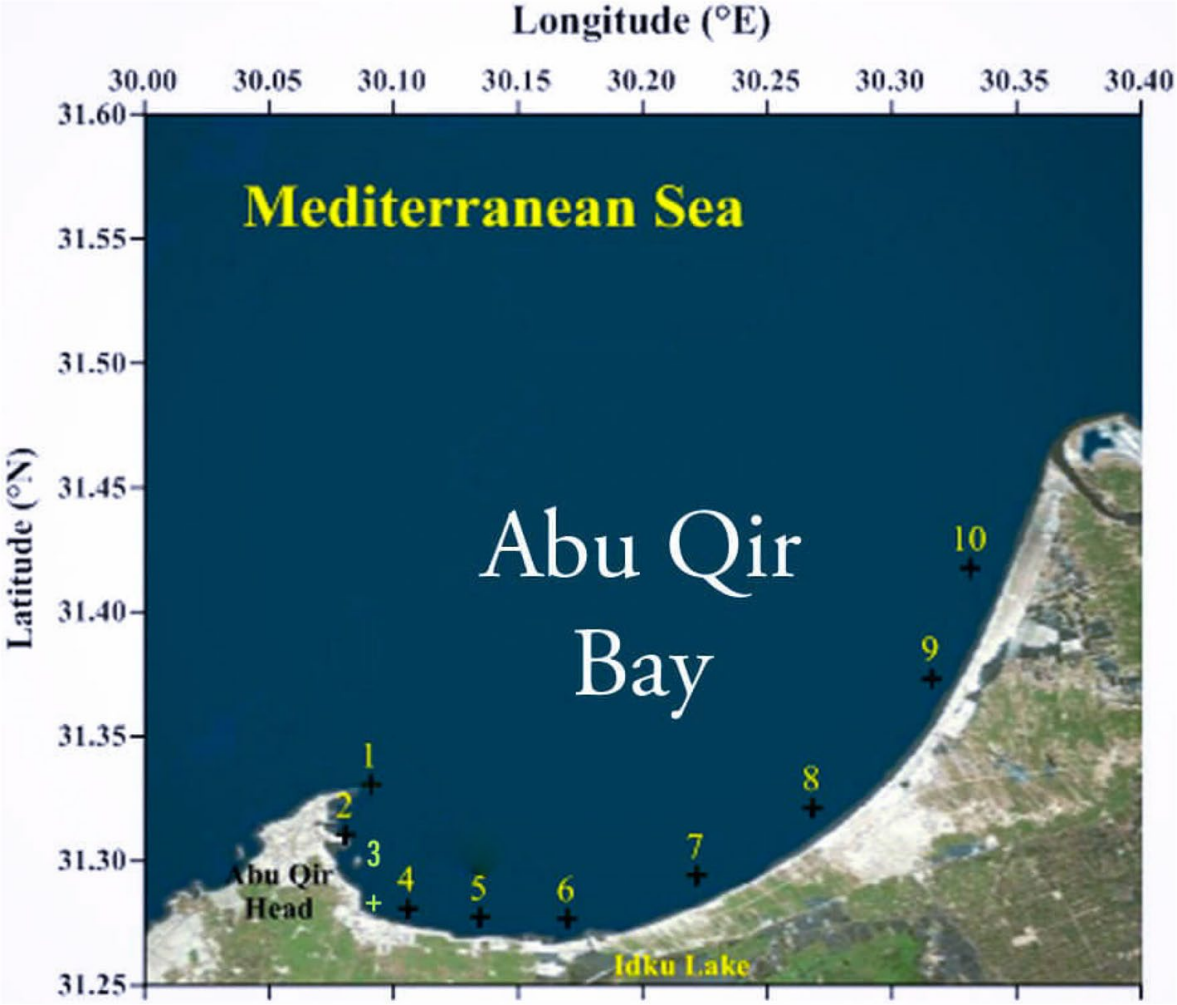
Geographic distribution of sampling sites

The tested actinobacteria were isolated from sediments during the summer of 2019, and the samples were collected along the Abu-Qir Bay to survey all the region located between the Rosetta region of the Nile and the Abu Qir town. Ten sediment samples were collected in sterile plastic containers under aseptic conditions from 5–20 cm below the surface of various locations in the Abu-Qir coast of the Mediterranean Sea to the west of Nile Delta in Egypt and were stored at 4 °C until being used (Fig. 1) [6].

### Samples treatment

**Fig. 2:**
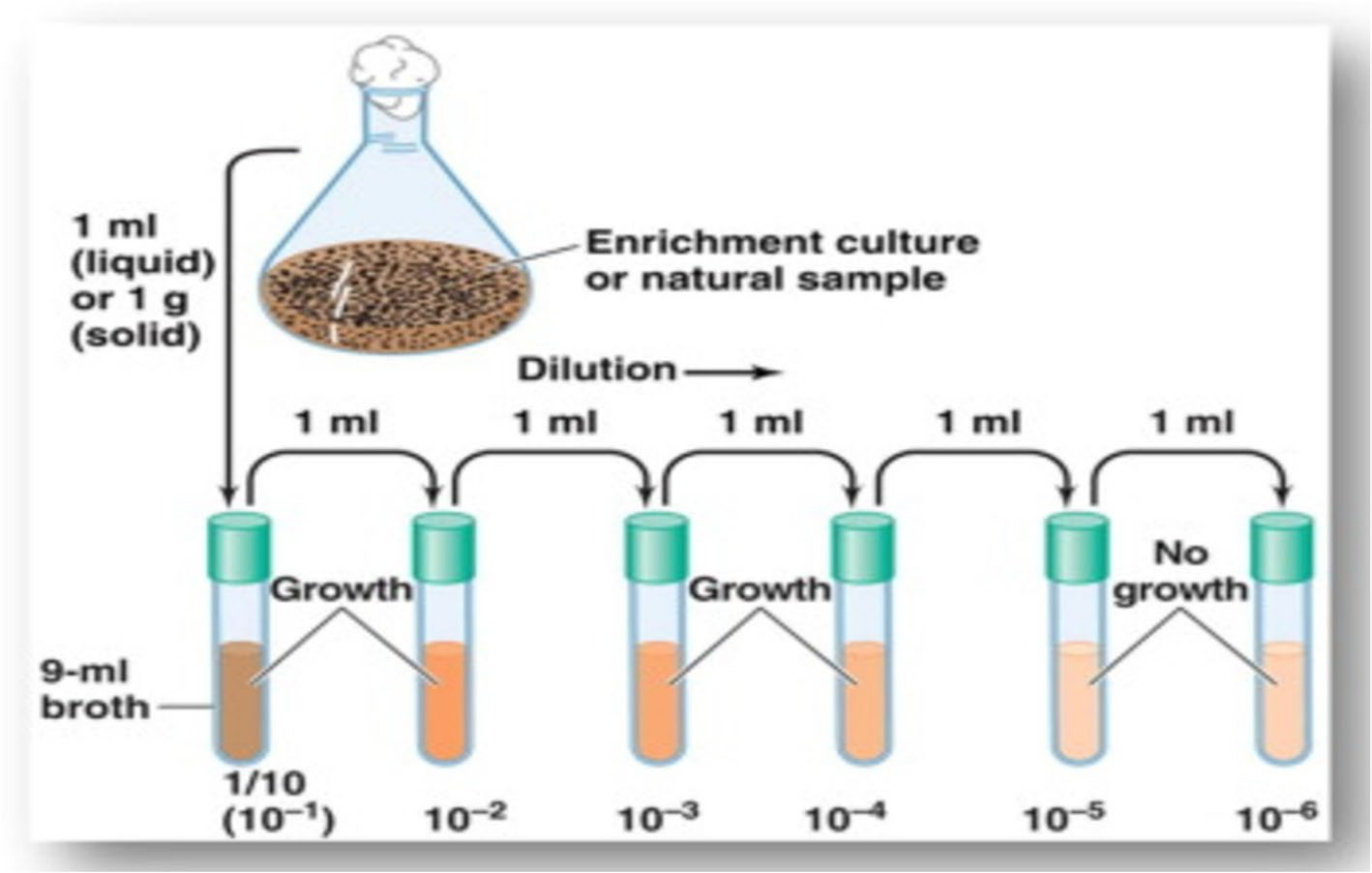
Serial dilution technique for microbe isolation

**Fig. 3:**
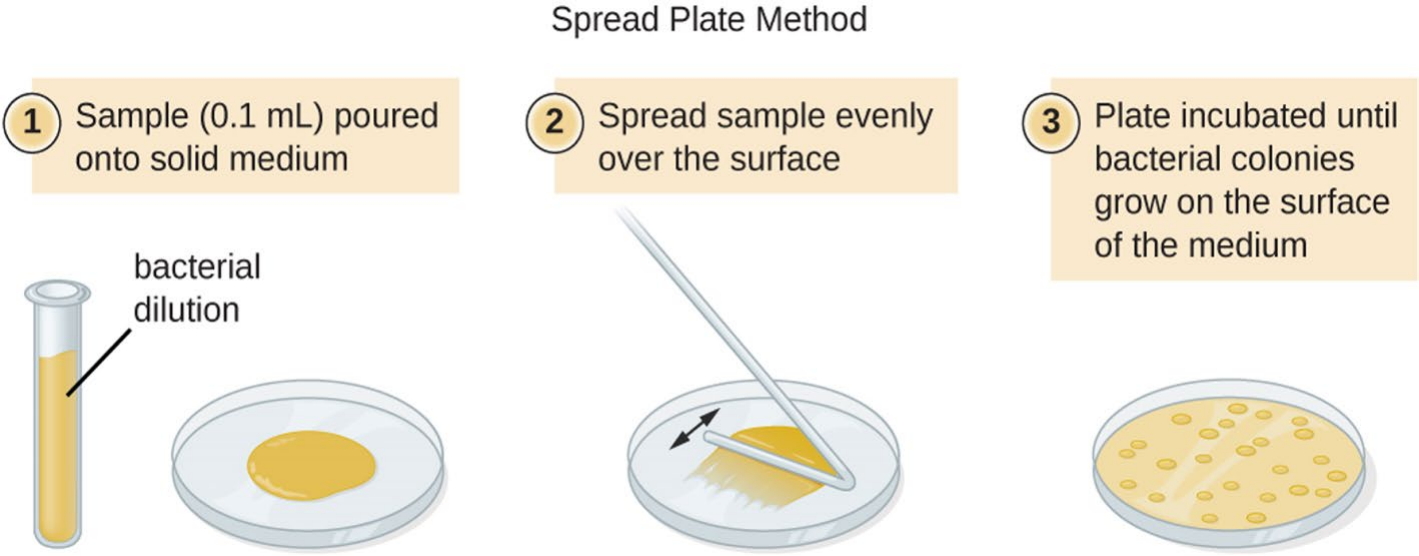
Pour plate method for microbe isolation

From each of the composite samples, 1 g of soil sample was added to a test tube containing 9-mL distilled water and shaked vigorously at room temperature (30 ± 2 °C), using an orbital shaker at 200 rpm for 10 min. The test tubes were considered as stock culture. Aseptically, 1-mL aliquot from the stock solution was transferred to a test tube containing 9 mL of sterile physiological saline and was mixed well. From these test tubes, 1 mL of aliquot was again transferred and mixed with another 9 mL of distilled water to make 10^−2^ dilution factor. Similarly, dilutions up to 10^−6^ were made using serial dilution technique (Fig. 2) for all samples. After serial dilution, 50 µL of each sample was separately plated using pour plate method (Fig. 3). The plates were incubated at 30 °C and observed from 3 to 14 days. The medium was supplemented with 75 and 25 μg ml^−1^ of filter-sterilized cycloheximide and nystatin respectively to minimize bacterial and fungal contamination [7].

### Isolation and identification of actinomycetes

#### Enumeration

The isolated actinomycetes were characterized by their features as aerial mycelia, spore formation, tough leathery colonies, and branched vegetative mycelia. Actinomycetes enumeration was done on starch nitrate agar, starch casino agar, glycerol asparagine agar, and glycerol glycine agar. Plates were incubated at 35 ± 2 °C for 7–14 days. Data are expressed as colony-forming unit (CFU)/g for dry-weight sediments [7].

#### Isolation and purification

The observed actinomycetes colonies from each sample were purified by repeated transfer on starch casein agar medium for growth until pure strains were obtained as judged by colony morphology. All isolates were grown at 35 ± 2 °C in 10 mL of starch casein broth, observed microscopically for the presence of mycelial fragmentation, and then frozen with 10% glycerol as a cryoprotectant [7].

### Media used for isolation and enumeration of actinomycetes

Starch nitrate medium contained (g/L) the following: starch, 20 g; KNO_3_, 1 g; K_2_HPO_4_, 0.5 g; MgSO_4_.7H_2_O, 0.5 g; FeSO_4_.7H_2_O, 0.01 g; and agar, 20 g. Starch casein medium (SCA) contained (g/L) the following: starch, 10 g; casein 0.30 g; KNO_3_, 2 g; K_2_HPO_4_, 2 g; MgSO_4_.7H_2_O, 0.05 g; FeSO_4_.7H_2_O, 0.01 g; CaCO_3_,0.02 g; and agar, 18 g. Glycerol asparagine contained (g/L) the following: L-asparagine, 1.0 g; glycerol, 10.0 g; K_2_HPO_4_.7H2O, 1.0 g; and agar, 20.0 g. Glycerol glycine contained (g/L) the following: glycerol, 20 g; L-glycerin, 2.5 g; K_2_HPO_4_, 1 g; MgSO_4_.7H_2_O, 0.1 g; FeSO_4_.7H_2_O, 0.01 g; CaCO_3_, 0.1 g; and agar, 20 g and then autoclaved for 20 min at a pressure of 1.5 lb/inch^2^ to raise the temperature to 121 °C, unless otherwise stated. pH was adjusted to 7 before sterilization, and all the chemicals were very pure from companies (Merck, Darmstadt, Sigma-Aldrich, Germany) [7].

### Phenotypic identification

#### Morphological characterization and pigmentation

Aerial hyphae, substrate mycelia, diffusible pigments, and the presence of colored spore mass on the surface of the colonies were examined visually for cultures (7–14 days old) grown on different media for morphological characterization [8].

##### Spore chain morphology

Starch casein medium was prepared, sterilized, and poured into petri dishes, and a coverslip was inverted at an angle of about 45° into each plate with the semisolid medium. Mycelial or spore suspensions were inoculated onto the connection between the starch casino medium and the coverslip’s upper surface. After 7–14 days of incubation at 35 ± 2 °C, the coverslip was carefully removed and placed onto a microscope slide for examination by a light microscope. Spore mass color could be categorized visually as gray, red, green, or other colors (white, yellow, blue, or violet) [8].

##### Substrate mycelium color

The cultures were grown at 35 ± 2 °C for 7–14 days on a starch casein medium. The confluent growth-bearing segments were removed from the inoculated plates, and the inverted and excess medium was removed from them to expose the substrate growth. An inverted segment was then placed at the end of a slide, and their reverse color was determined visually [8].

### Biochemical and physiological analysis

#### Effect of incubation temperatures on the growth of the isolated actinomycetes

The tested isolates were examined for their ability to grow at different incubation temperatures (25, 30, 40, and 50 °C) using starch casein agar medium for 7 days at pH 7; the growth was estimated visually at the end of the incubation period [9].

#### Effect of the initial pH on the growth of the isolated actinomycetes

Optimal pH was determined by growing the tested isolates on starch casein agar medium and adjusted to different pH values (5–9); at the end of the incubation period, the growth was estimated visually by the presence of substrate and aerial mycelia [9].

### Carbon source utilization

Different carbon source solutions (w/v) (glucose, fructose, maltose, lactose, and mannitol) were sterilized by filtration. Each of the tested sterilized carbon sources was added aseptically at a concentration of 1% (w/v) to the basal mineral salts agar medium. pH was adjusted to 7.0. After autoclaving, the basal medium was cooled at 60 °C and then fortified with sterile carbon sources with the final concentration required. The medium was agitated and poured (20 mL) into petri dishes. Duplicate plates for each culture were prepared. The plates were left for solidification. The standard inoculum of the actinomycetes under test was streaked over the surface of the agar plates, incubated at 35 ± 2 °C for 7 days and pH 7. At the end of the incubation period, the growth was recorded visually by the presence of substrate and aerial mycelia [9, 10].

### Sodium chloride tolerance

Actinomycetes media (SCA) was prepared with distilled water in 5 different sets which were supplemented with different concentrations of NaCl (g/L): 0, 40, 70,100, and 130 one at a time, autoclaved, and poured into sterilized plates which were inoculated with the tested strains. Observations were made after 7 days of incubation at 35 ± 2 °C. The highest concentration of salt that allowed growth was recorded. There was no clear-cut borderline between growth and no growth since in most cases growth was retarded and or less dense in the form of smaller colonies, with higher salt concentrations tolerated as compared with the control [9, 10].

### Protease production

Skim milk agar medium containing 3% agar and 2% skim milk was incubated at 35 ± 2 °C for 7 days, a clear area around the growth of the culture referred to positive results [11].

### Lipase production

Degradation of Tween 20 (1% w/v) was detected on lipolytic activity medium. After incubation at 35 ± 2 °C for 7 days, clearing of insoluble components under and around the areas of growth was scored as a positive result [12].

### Urease production

Urea broth basal medium was prepared and dispensed in the test tubes 9 mL each, and then the tubes were inoculated and incubated at 35 ± 2 °C for 7 days. The appearance of a purple-pink color indicated the production of ammonia, i.e., urea was decomposed (positive test) [13].

### Catalase production

An organism is tested for catalase production by the addition of 3% hydrogen peroxide to 7-day-old culture. Bubbles of oxygen are released if the organism is a catalase producer [14].

### Chitinase production

Degradation of chitin (colloidal, 0.25% w/v) was detected on chitinolytic activity medium (P.25). After incubation at 35 ± 2 °C for 7 days, clearing of insoluble components under and around the areas of growth was scored as positive results [15].

### Cellulase production

Cellulose (CMC) degradation was tested using a cellulolytic activity medium after 7 days at 35 ± 2 °C, and clearing of insoluble compounds from under and around areas of growth was scored as positive [16].

### Gelatinase production

Degradation of gelatin was tested using gelatin medium. After incubation at 35 ± 2 °C for 7 days, the plates were flooded with mercuric chloride solution (mercuric chloride, 15 g; concentrated hydrochloric acid, 20 ml; water 100 ml). The appearance of clear zones around the gelatin liquefying colonies was scored as a positive result [17].

### Clustering of the isolates relationships

Clustering was performed to group similar strains together. A commonly used clustering algorithm is hierarchical clustering, or HCL, and the resultant Newick trees were then displayed by MEGA version 4.0.2 and added to the final revised manuscript.

### Molecular identification

For molecular identification, sequencing of 16S rRNA was used as a tool for genotypic characterization. This step was performed using a 3730 automated DNA sequencer at Sigma for scientific research. A simplified rapid protocol for preparing DNA from the isolates was used in this work. A polymerase chain reaction (PCR) was done to amplify the 16S rDNA genes from the promising actinomycetes genomes using universal primers designed to amplify ~1500 bp of this gene. The forward primer was 5′-AGAGTTTGATCMTGGCTCAG-3′ (position 8 in the 16S rRNA gene according to *E. coli* numbering), and reverse primer was 3′-TACGGYACCTTGTTACG ACTT-5′ (position 1514 in the 16S rRNA gene according to *E. coli* numbering), which were then sequenced, and the BLAST program was used to assess similarity [6].

### Plackett–Burman design [18]

Application of the statistical design was carried out in a “two-phase” optimization approach. The first step was to evaluate the relative importance of the various constituents in the culture media and select levels of variables that have a significant influence on pigment production. The second was the verification of the experiments to validate results under specific optimized experimental conditions. The Plackett–Burman experimental design, a fractional design, cannot determine the exact quantity but can provide indication and tendency regarding the necessity of each factor in relatively few experiments. Therefore, the aim of the following experiments was to optimize medium composition for maximum pigment production and to evaluate the factors significantly affecting pigment production, using Plackett–Burman experimental design. For each nutrient variable, a high (+) or low (−) concentration was tested. After applying the ANOVA statistical test, it was found that the first-order models for pigment production were satisfactory, and the linear model equation was proposed to calculate the optimum levels of these variables for pigment production can be written as follows:

$$Y=0.010978-0.00025\times \mathrm{starch}-0.003\times \mathrm{KNO}3-0.002825\times \mathrm{K}2\mathrm{HPO}4-0.001025\times \mathrm{MgSO}4+0.003\times \mathrm{FeSO}4+0.0052\times \mathrm{casein}+0.000275\times \mathrm{CaCO}3$$

where Y represents pigment production in g/L.

### Validation of the model

A pre-optimization step should be done for the subsequent optimization step. In this step, a pre-optimization formula was prepared, where the most significant variables were fixed at their optimum levels obtained from the Plackett–Burman design. On the other hand, the other variables with a negative effect value were fixed at their “−1” coded values, and those with a positive effect value were fixed at their “+1” coded values. The purpose of this step was to confirm the results of the Plackett–Burman design and to construct the basic formula for further optimization step.

### Response surface methodology [19]

After estimating the relative significance of independent variables, the most significant variables were selected for further determination of their optimal level with respect to antimicrobial activity. For this reason, the Box-Behnken design, which is a response surface methodology, was applied. This optimization process includes three main steps:

- Performing the statistically designed experiments
- Estimating the coefficients in a mathematical model
- Predicting the resulted response, and the model adequacy was checked.

The significant variables identified by the Plackett–Burman design were further optimized by response surface methodology using the Box-Behnken experimental design. The three key variables (casein, KNO_3_, and FeSO_4_) were examined at three different levels −, 0, and + for maximal pigment production. A design matrix for 15 trials for maximal pigment production, along with the natural values for the factors, was constructed.

By applying the analysis of the quadratic regression to the experimental data, this equation was applied to explain pigment production:

$${\varvec{Y}}=-0.16+0.04\times {\mathbf{X}}_{1}+0.22\times {\mathbf{X}}_{2}+9.83\times {\mathbf{X}}_{3}-0.36\times {\mathbf{X}}_{1}\times {\mathbf{X}}_{2}+1.2\times {\mathbf{X}}_{1}\times {\mathbf{X}}_{3}-0.28\times {\mathbf{X}}_{2}\times{\mathbf{X}}_{3}+0.47\times {\mathbf{X}}_{1}^{2}+0.03\times {\mathbf{X}}_{2}^{2}-80\times {\mathbf{X}}_{3}^{2}$$

where Y is the dependent variable (pigment production) in g/L and X_1_, X_2_, and X_3_ are the concentrations of the independent variables

Normally, a regression model having an *R*^2^ value higher than 0.9 is considered as having a very high correlation, and a model with an *R*^2^ value between 0.7 and 0.9 is considered as having a high correlation.

### Verification of the optimized results

The optimal conditions obtained from the optimization experiments were verified experimentally and were then compared to the data calculated from the model.

### In vitro inhibition of albumin denaturation assay [20]

The anti-inflammatory activity was studied using the inhibition of albumin denaturation technique with minor modifications. A total of 2.25 mL of bovine serum albumin (BSA) (1%, w/v aqueous solution) was added to 25 mL of the samples (the pigment extracts solutions). Blank and standard solution (diclofenac sodium “Voltaren® ampoule Novartis Pharma” 1000 μg/mL) were adjusted to pH, 5.5. The samples were incubated at 37 °C for 20 min and then transferred to 60 °C water bath for 5 min. Following incubation, the samples were left to cool down at room temperature, and then 2.5 mL of phosphate buffer was added to the above solutions. The absorbance of the above solutions was measured using UV-Visible spectrophotometer at 660 nm (Unico-UV Visible Spectrophotometer Model UV-2000, USA). The percentage of inhibition of protein denaturation was calculated using the following formula:

Inhibition of protein denaturation (%) is as follows.

$$=\left(\frac{\mathrm{absorption\, of\, control}-\mathrm{absorption\, of\, test}}{\mathrm{absorption\, of\, control}}\right)\times 100$$

The control represents no inhibition of protein denaturation.

Each of the tested supernatant activities was compared with the most commercial standard anti-inflammatory agent “diclofenac sodium”.

## Results

### Isolation and enumeration of actinobacteria

**Fig. 4:**
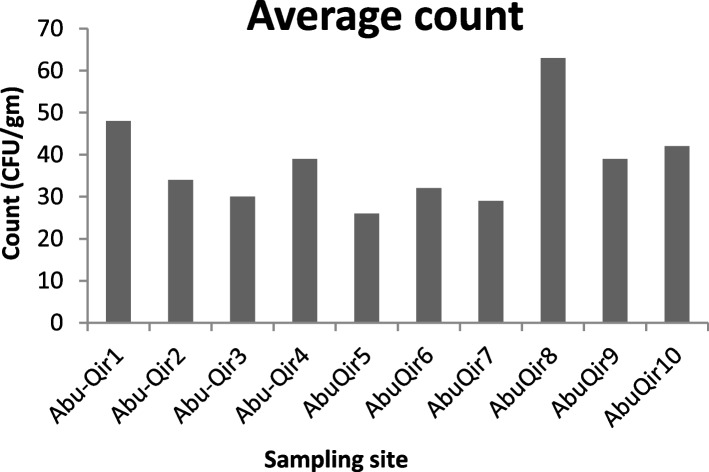
Average viable count of actinomycetes at different sampling sites

**Fig. 5:**
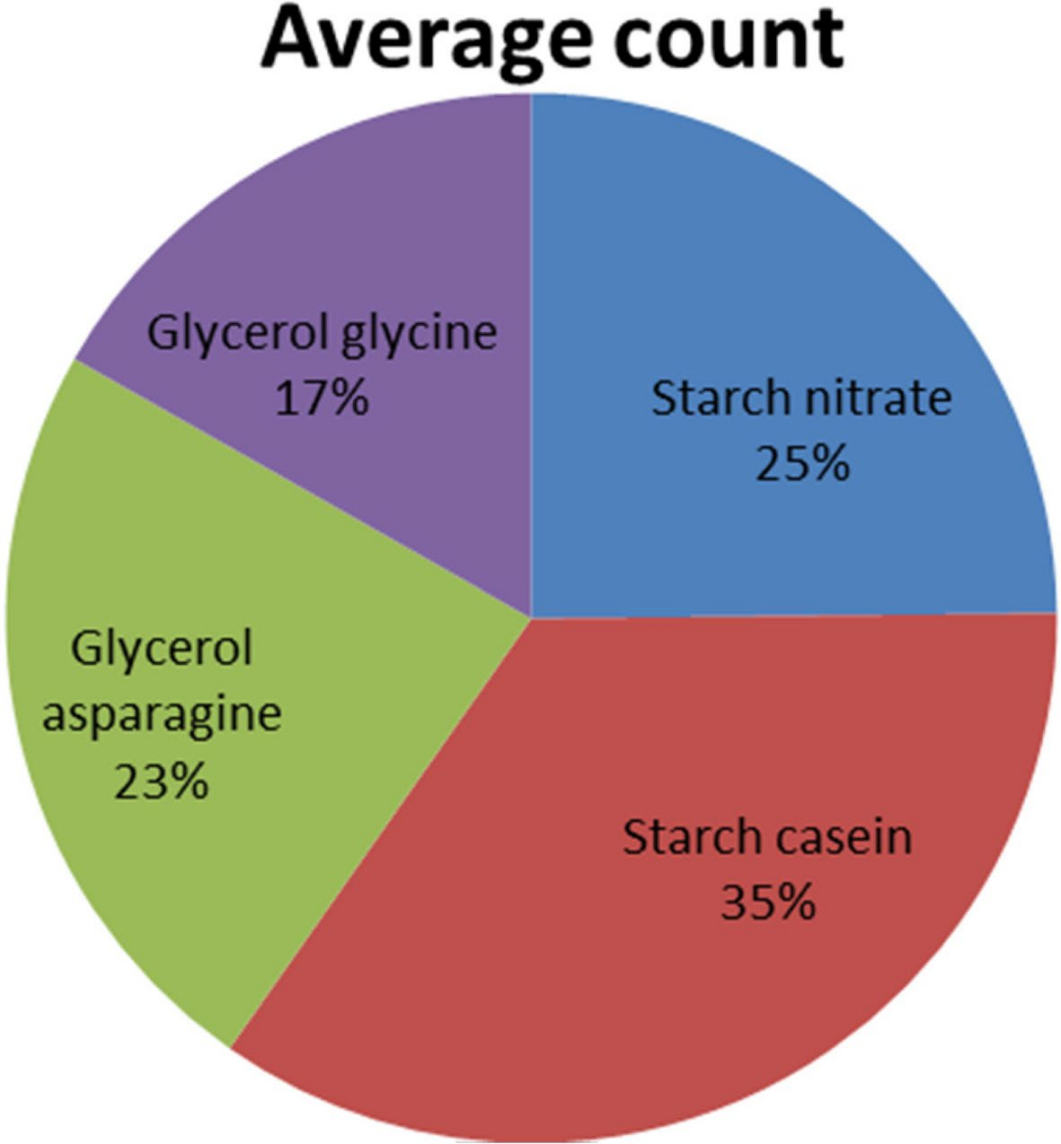
Average viable count of actinomycetes on different media

Sediment samples were collected during summer of 2019 from 10 different sites as shown in Fig. 4. Four different media, namely starch nitrate, starch casein, glycerol asparagine, and glycerol glycine, were used as a preliminary experiment to elucidate the role of the medium components on the actinomycetes counts in sediment samples and to select the one that allows the development of the highest counts. As clearly observed in Table 1 and Fig. 5, starch casein medium reported the highest counts (30–63 CFU/g) in all the tested sites. Lower counts were detected on glycerol asparagine and glycerol glycine. On the other hand, glycerol glycine medium harbored the lowest counts (15–48 CFU/g). Abu-Qir_8_ harbored the highest average count of actinomycetes (63 CFU/g), followed by Abu-Qir_1_ (48 CFU/g). The lower counts were detected in Abu-Qir_5_ and Abu-Qir_7_ (26 and 29 CFU/g, respectively).

**Table 1:** Viable counts of actinomycetes as affected by media composition at Abu-Qir Bay sediment samples (CFU/g)

Sampling sites	Actinomycetes count (CFU/g)				
Media	Starch nitrate	Starch casein	Glycerol asparagine	Glycerol glycine	Average count
Abu-Qir_1_	48.0	62.0	54.0	26.0	48.0
Abu-Qir_2_	32.0	42.0	36.0	25.0	34.0
Abu-Qir_3_	31.0	36.0	32.0	20.0	30.0
Abu-Qir_4_	40.0	44.0	42.0	28.0	39.0
AbuQir_5_	22.0	42.0	21.0	17.0	26.0
AbuQir_6_	34.0	54.0	23.0	16.0	32.0
AbuQir_7_	24.0	30.0	18.0	15.0	29.0
AbuQir_8_	60.0	90.0	54.0	48.0	63.0
AbuQir_9_	38.0	52.0	34.0	30.0	39.0
AbuQir_10_	44.0	63.0	32.0	28.0	42.0
Average count	37.0	52.0	35.0	25.0	38.0

### Clustering of actinomycetes isolates using the neighbor-joining method

#### Numerical taxonomic analysis

**Fig. 6:**
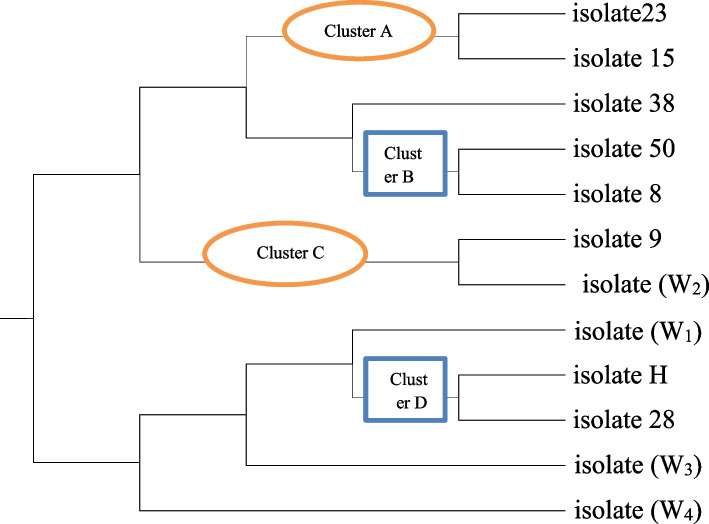
Simplified dendogram for cluster relationships

A total of 12 pure isolates were subjected to morphological, physiological, and biochemical characterization. The distribution of positive characters was shown in Tables 1S, 2S, 3S, and 4S. The selected actinobacterial isolates were subjected to numerical analysis. Data of the dendogram (Fig. 6) revealed that at 83% similarity level, the majority of isolates were grouped into four main clusters (A, B, C, & D), each of them harbored two isolates, and additionally, four isolates did not cluster at this similarity level. Cluster A as shown in Table S1 contained two isolates (23 & 15). Both isolates were well developed on all tested media except yeast malt extract agar. Oatmeal was utilized by isolate 15 only. Isolate 15 was characterized by grayish-yellow substrate mycelium and yellow aerial mycelium, and isolate 23 showed yellowish-white substrate mycelium and gray aerial mycelium. No diffusible pigments were detected. The optimum growth temperature range of the two isolates was 25–40 °C at pH range 7–9 and extended up to 50 °C with pH 5–9 for isolate 15. Both isolates tolerated up to 10% NaCl, and NaCl was obligated for the growth of isolate 23. Both isolates could utilize all the tested sugars with the production of proteases, lipases, catalases, chitinase, and cellulases, but they were not able to synthesize ureases or gelatinases. Cluster B as shown in Table 2S harbored two isolates (50 & 8). Both of them grew well on all media except isolate 8 that could not grow on oatmeal agar. Both of them had grayish-yellow substrate mycelium with cream aerial mycelium. No diffusible pigments were detected. Also, they well developed at the temperature range 25–50 °C. The optimum growth pH levels ranged from 5 to 7. Isolate no. 50 was able to grow in the presence of NaCl up to 7%, while isolate 8 was unable to grow in the absence of NaCl and was able to tolerate the presence of NaCl up to 10%. Both isolates were able to utilize all the tested sugars with the production of proteases, lipases, ureases, chitinases, and cellulases; no catalases or gelatinases were detected in the cultures of both isolates. Cluster C as shown in Table 3S contained two isolates (9 & W2). Both isolates grew well at all tested media at the temperature range 25–50 °C and pH range 5–9. Both isolates had a violet substrate mycelium with a diffusible violet pigment. Isolate W2 had pale violet aerial mycelium, while isolate 9 had white aerial mycelium. Isolate 9 was able to tolerate NaCl up to 10%, while isolate W2 was able to tolerate up to 7% NaCl. Both isolates were able to utilize all the tested sugars with the production of all enzymes except gelatinases.

#### Cluster D

As shown in Table 4S, cluster D contained two isolates (H & 28). Both isolates grew at all media, but isolate 28 could not grow at Czapek Dox Agar. The substrate mycelia were brown, while the aerial mycelia were white for both isolates. They had the ability to produce brown diffusible pigments. They grew well at a temperature range of 25–40 °C and a pH range 5–9. Isolate H was able to tolerate up to 10% NaCl, while isolate 28 was able to grow in the presence of 4–7% NaCl. Both isolates were able to utilize all the tested sugars with the production of proteases, lipases, chitinases, and cellulases, while urease was only produced by isolate 28. Both isolates lack the ability to produce catalases and gelatinases.

#### Single clusters

There were four isolates formed in four single clusters as shown in Table 5S, and isolate was 38 able to grow well on all the tested media. Yellowish-white substrate mycelium with yellow aerial mycelium was detected. No diffusible pigment was produced. The growth temperature ranged from 25 to 40 °C at a pH range 5–9. It was able to utilize all tested sugars. It was able to tolerate up to 10% NaCl. All enzymes, protease, lipase, chitinase, urease, and cellulase, were produced by the tested isolate except catalase and gelatinase; also, isolate W1 was able to grow on all the tested media with brown substrate mycelium and white aerial mycelium. It was able to produce beige to brown diffusible pigments. The optimum growth temperature range was 25–50 °C at a pH range 5–9. All sugars were utilized, and NaCl was tolerated up to 10% by the tested isolate. Isolate W1 was able to synthesize protease, lipase, chitinase, cellulase, catalase, and gelatinase, while it was not able to produce urease enzyme. But isolate W3 was able to grow only on three tested media (inorganic salts starch agar, oatmeal agar, and starch nitrate agar), while it could not grow on the other three tested media (yeast malt extract agar, Czapek Dox Agar, and Krassilnikov agar). Creamy substrate mycelium and white aerial mycelium were detected, but no pigment was detected. The optimum growth temperature range was 25–50 °C at a pH range 5–9. It was able to utilize all the tested sugars and tolerate up to 7% NaCl. It was able to produce protease, lipase, chitinase, cellulose, and gelatinase, while it was not able to produce urease or catalase. Isolate W4 had the ability to grow on all the tested media except the Krassilnikov agar medium. Green substrate mycelium and yellow aerial mycelium were detected. It was able to produce green diffusible pigment. The growth temperature range was 25–50 °C at a pH range 5–9. It was able to utilize all the tested sugars, and it was able to tolerate up to 7% NaCl. All enzymes were produced (protease, lipase, urease, catalase, chitinase, cellulose, and gelatinase).

### Identification and characterization of the selected isolate (W4)

**Fig. 7:**
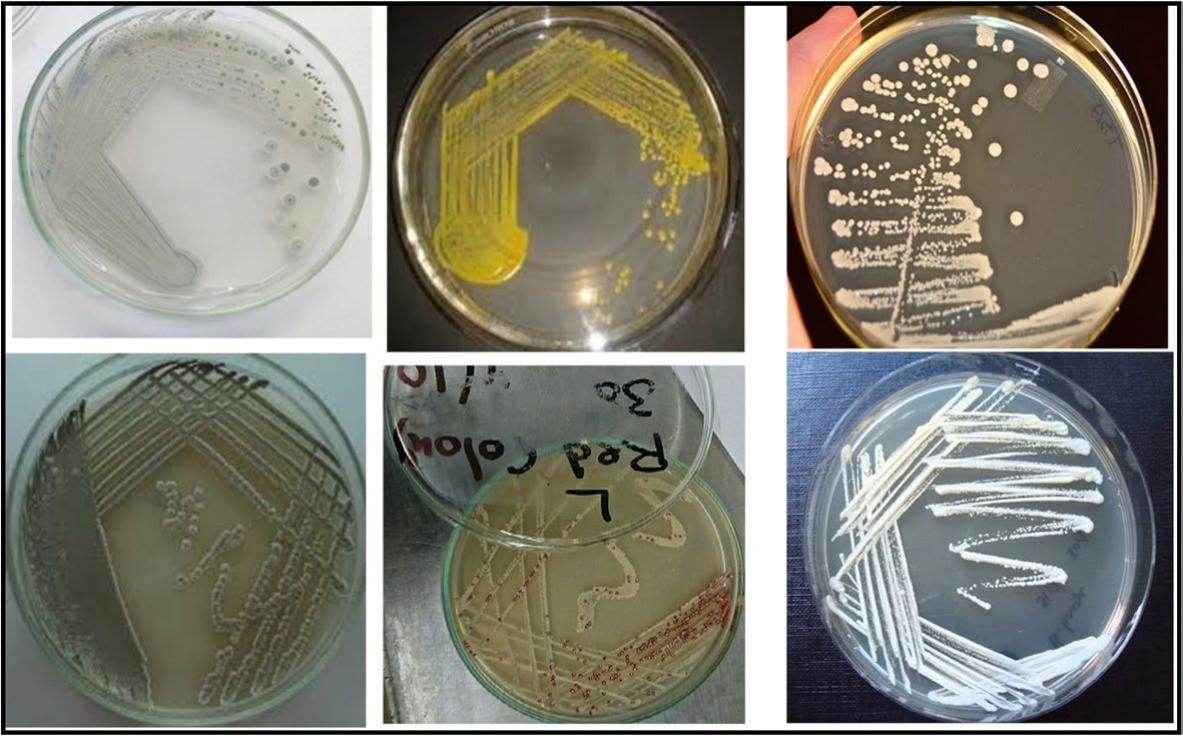
Isolation of Actinomycetes like cells and purification of some represented isolates

**Fig. 8:**
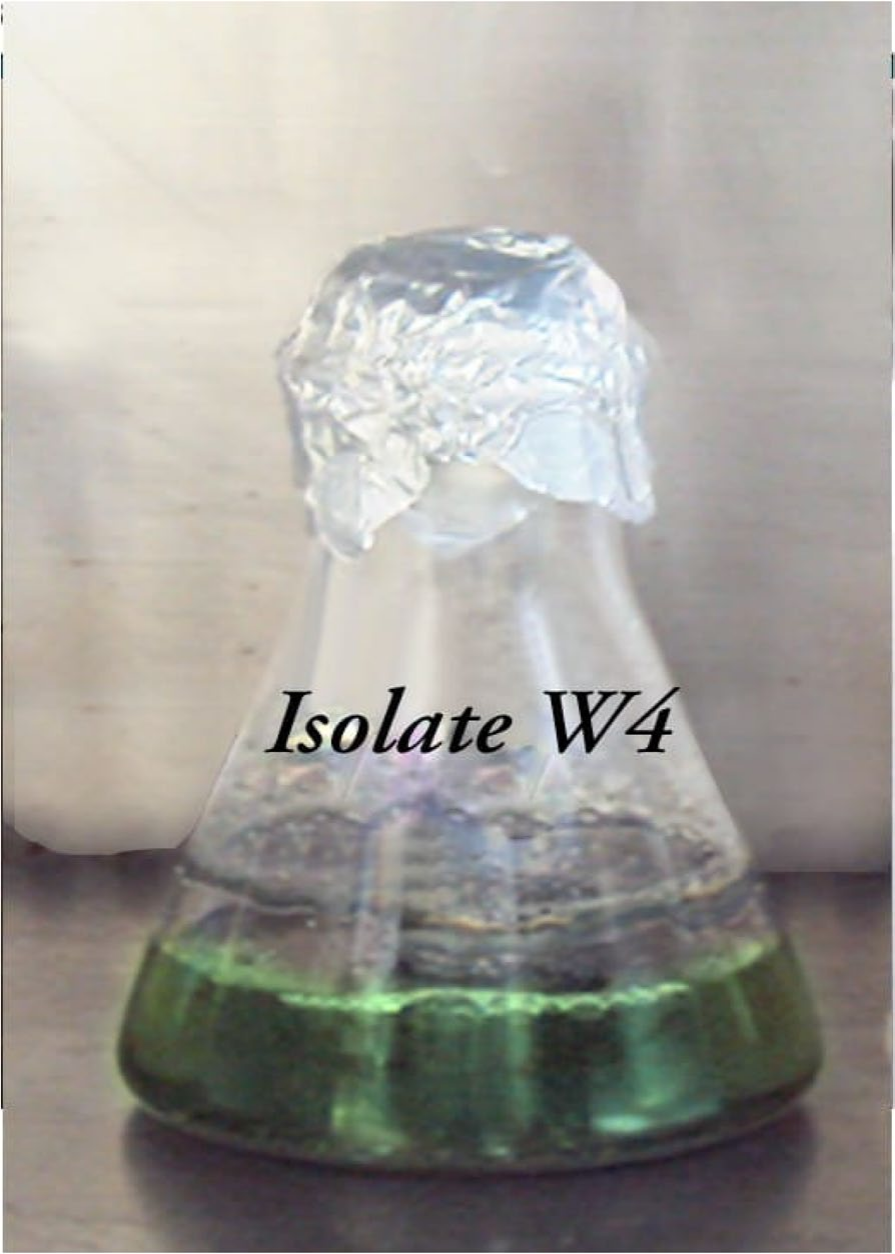
Green pigmented broth of the *Streptomyces tunisiensis* W4MT573222

Based on the results of numerical taxonomic analysis, clustering, and antagonistic interactions, isolate W4 was carefully chosen as the most promising pigment and antimicrobial agent’s producer as Ibrahim et al. [6], and it has the ability to produce the unique obvious green pigment and also able to inhibit the growth of most of the pathogens. For these reasons, it was carefully chosen for molecular phylogenetic analysis, identification, and scanning electron microscope as previously mentioned (Figs. 7 and 8) [6].

### Screening of significant variables affecting pigment production by *Streptomyces tunisiensis* W4MT573222 using Plackett–Burman experimental design

**Fig. 9:**
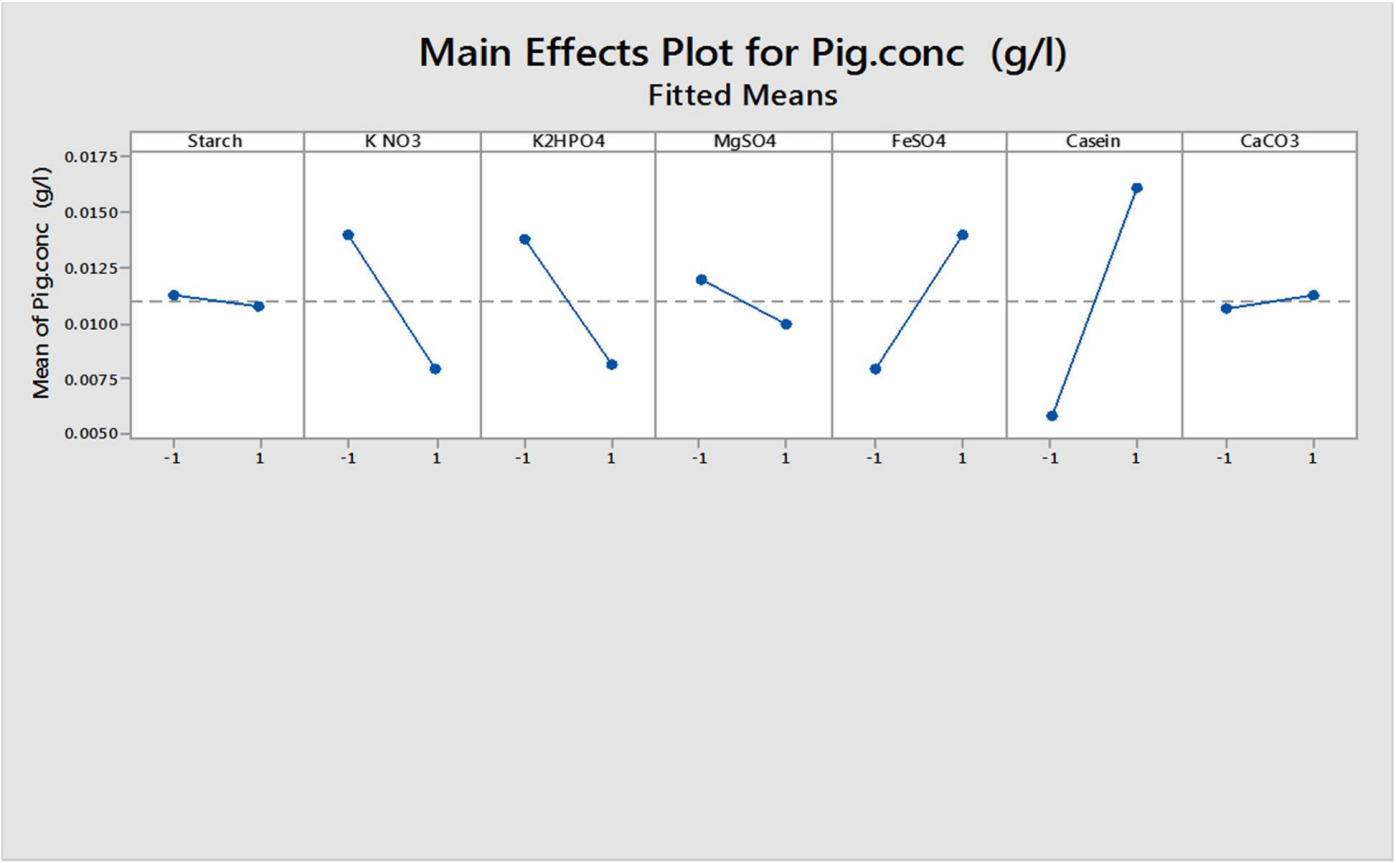
The main effect of different variables on pigment production, based on the results of Plackett–Burman design

**Fig. 10:**
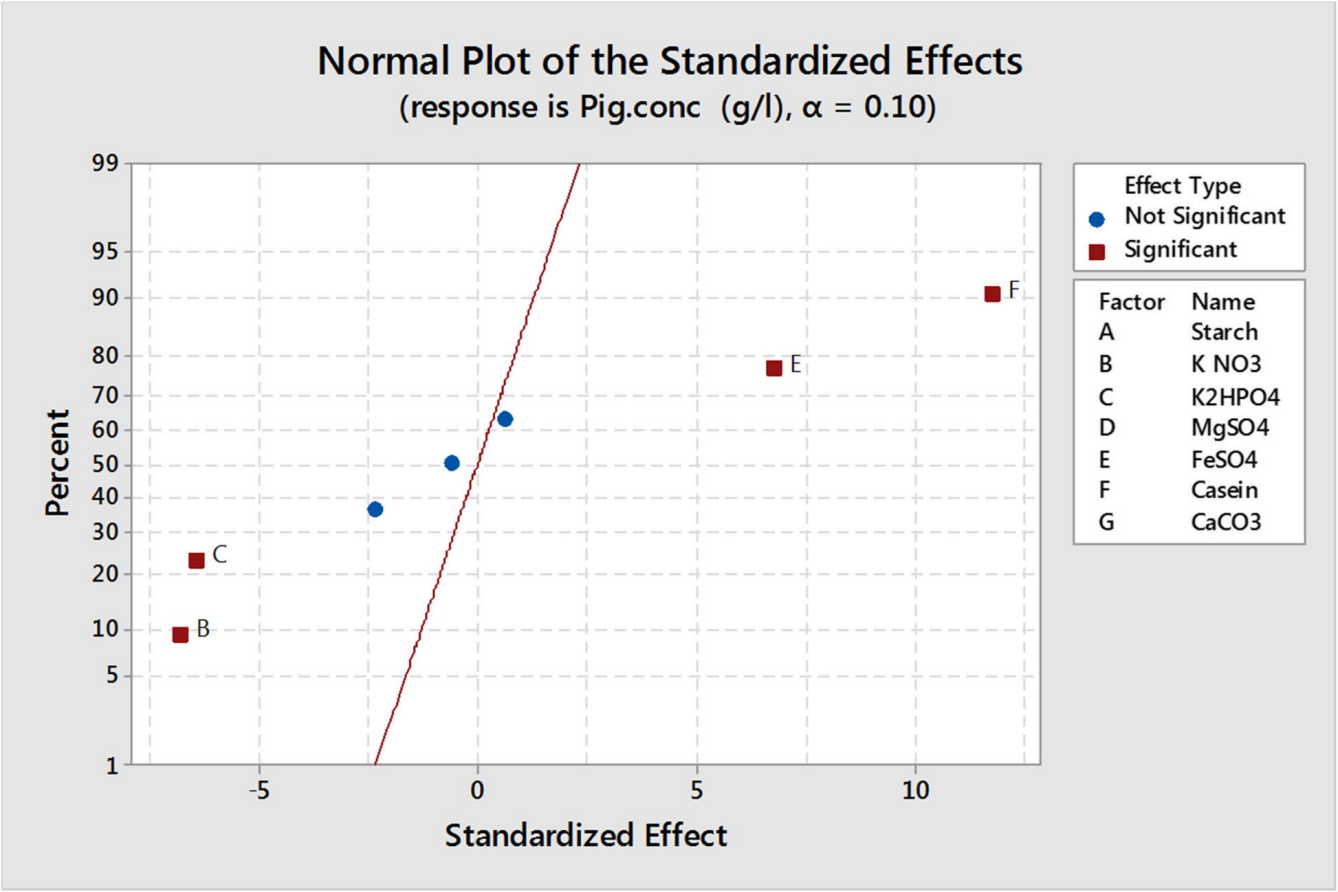
Effect of different nutrient variables on pigment production validated by statistical analysis, normal plot of the seven variables showing the significant variables in red, and the nonsignificant variables in blue

The Plackett–Burman experimental design is a fractional design, which is unable to examine the precise quantity but can offer tendency and indication regarding the importance of each variable in relatively limited experiments. Therefore, the main target of the following experiments was the optimization of the composition of the culture medium for optimizing the produced pigment yield and to evaluate the significantly affecting factors on the pigment production, using PBD. The PBD for seven different variables with two levels of concentrations in Table 6S was carried out in eight trials according to the design of the experimental matrix shown in Table 2. The results obtained are listed in Table 2, where the pigment production ranged from 0.0012 to 0.0241 g/L. These data were used to compute the main effect of the independent variables, and the results are presented graphically in Fig. 9. The high concentration of each tested variable indicated by a positive sign main effect is near to the optimum value in contrast to the tested factor low concentration indicated by the negative sign main and is near to optimum, but a mean close to zero indicates that a variable has no or little effect. Table 7S shows the Plackett–Burman analysis, and it was found that starch, KNO_3_, K_2_HPO_4_, and MgSO_4_ had a negative effect on pigment production within the assessment ranges, while FeSO_4_, casein, and CaCO_3_ had a positive effect on pigment production. The highest improvement percentages of 146, 106, 27.6, and 5.1% were detected in trial numbers 1, 2, 8, and 3, respectively (Fig. 10). The *t*-test for each individual effect permits the probability evaluation of the observed effect only by chance. The statistical confidence was calculated as follows:

**Table 2:** Plackett–Burman design matrix for seven variables with coded levels

Trial	Starch	KNO_3_	K_2_HPO_4_	MgSO_4_	FeSO_4_	Casein	CaCO_3_	Pig. conc (g/L)	Percentage of improvement in pigment production (%)
1	−1	−1	−1	1	1	1	−1	0.0241	145.9
2	1	−1	−1	−1	−1	1	1	0.0202	106.1
3	−1	1	−1	−1	1	−1	1	0.0103	5.1
4	1	1	−1	1	−1	−1	−1	0.0012	−87.8
5	−1	−1	1	1	−1	−1	1	0.0026	−73.5
6	1	−1	1	−1	1	−1	−1	0.0096	−2.0
7	−1	1	1	−1	−1	1	−1	0.0085	−13.3
8	1	1	1	1	1	1	1	0.0125	27.6
9	0	0	0	0	0	0	0	0.0098	0.0

$$\mathrm{Statistical\, confidence}=(1-p)\times 100.$$

**Fig. 11:**
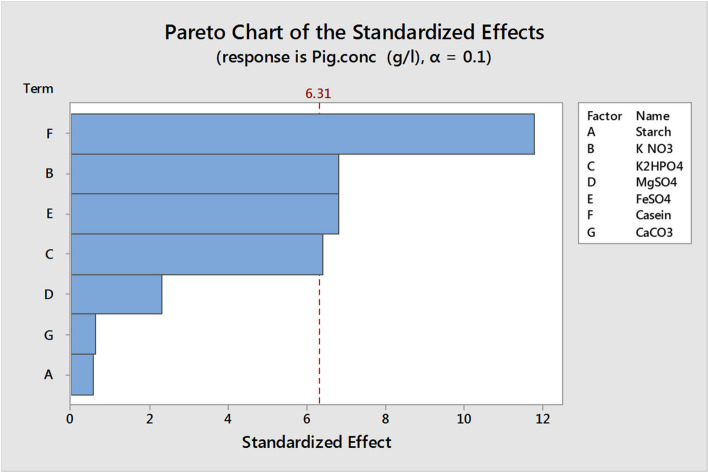
Pareto chart of Plackett––Burman showing the effect of media components on pigment production using *Streptomyces tunisiensis* W4MT573222. The vertical line indicates significant level of 90% for the effects

Hence, any component showing statistical confidence higher than 90% (*P* = 0.1) was considered significant. Based on the statistical analysis of the confidence level of seven variables (Table 7S), KNO_3_, K2HPO_4_, FeSO_4_, and casein had confidence levels greater than 90% and hence were considered the significant parameters which influence pigment production in Fig. 11. Figure 11 shows the ranking of factor estimates in a Pareto chart. The Pareto chart has been used as a useful identifying tool for the most important effects. It shows each variable magnitude and is a suitable way to show the results of a Plackett–Burman design. In this chart, the length of each bar on a standardized Pareto chart is proportional to the absolute value of its associated regression coefficient or estimated effect. The fit goodness of the model was checked by the coefficient determination (*R*^2^). In this study, the *R*^2^ value was measured to be *0.9964*, indicating that 99.64% of the response total variability could be described by this design, and only 0.36% of the variation was not described. After using the ANOVA statistical test, it was found that the pigment production first-order models were acceptable; the linear model equation was proposed to measure the optimum levels of these factors for pigment production can be written as follows:

$$Y=0.010978-0.00025\times \mathrm{starch}-0.003\times \mathrm{K N}{\mathrm{O}}_{3}-0.002825\times \mathrm{K }2{\mathrm{HPO}}_{4}-0.001025\times {\mathrm{MgSO}}_{4}+0.003\times {\mathrm{FeSO}}_{4}+0.0052\times \mathrm{casein}+0.000275\times {\mathrm{CaCO}}_{3}$$

where Y represents pigment production in g/L.

**Fig. 12:**
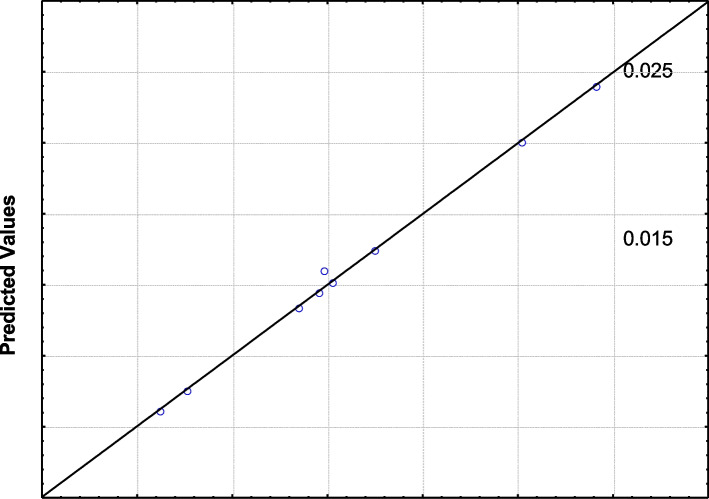
Correlation between predicted and observed values in Plackett–Burman

The Plackett–Burman design can be used to predict pigment production within the limits of the experimental factors. Figure 12 shows that the predicted response values agreed well with the actual response values. The nearby correlation between the actual and predicted data showed the model’s appropriateness.

### Validation of the model

Depending on the obtained data from PBD results, the following composition (g/L) was predicted to be near optimum: starch, 5 g; KNO_3_, 1 g; K_2_HPO_4_, 1 g; MgSO_4_, 0.025 g; FeSO_4_, 0.015 g; casein, 0.45 g; and CaCO_3_, 0.03 g, and the medium was adjusted at pH 7, and the flask was incubated at 37 °C on a rotary shaker at 120 rpm. The predicted near-optimum levels of dependent variables were examined and compared to the basal condition settings and pigment production. The applied near optimum condition resulted in pigment production of approximately 0.0467 g/L. This result represented a 4.8-fold increase in pigment production when compared with the obtained results average using the basal condition.

### Optimization for pigment production by Box-Behnken design

The significant factors so recognized by the PBD experiment were further optimized by response surface methodology (RSM) using the BB experimental design. The three key variables (casein, KNO_3_, and FeSO_4_) were studied at three different levels −, 0, and + (Table 8S) for maximal pigment production. Table 8S shows different combinations of the three chosen factors according to the BBD. The remaining component concentrations in all tested trials were the same concentration as those used in the pre-optimized medium of the PBD. The obtained data from the BB experimental design were indicated in Table 3. The maximal production of the pigment was 0.096 g/L in trial number 14, with a percentage improvement of 105.6 compared with the control (obtained from pre-optimized PBD), followed by trial numbers 3,12, 4,7, 15, and 9, which gave an improvement percentage of 40.1, 39.2, 32.3, 31, 22.5, and 9.2, respectively.

**Table 3:** Box-Behnken design matrix and results for the most significant three variables that affected pigment production (casein (X_1_), KNO_3_ (X_2_), and FeSO_4_ (X_3_))

Trial	Variable			
	X_1_	X_2_	X_3_	Pigment production (g/L)
1	0	0	0	0.0467
2	0	0	0	0.0467
3	0	−1	1	0.06542
4	0	1	−1	0.06178
5	−1	−1	0	0.0368
6	0	0	0	0.0467
7	1	0	−1	0.0612
8	0	1	1	0.0348
9	0	−1	−1	0.051
10	−1	0	1	0.04
11	−1	0	−1	0.0374
12	1	0	1	0.065
13	1	1	0	0.0438
14	1	−1	0	0.096
15	−1	1	0	0.0572

**Fig. 13:**
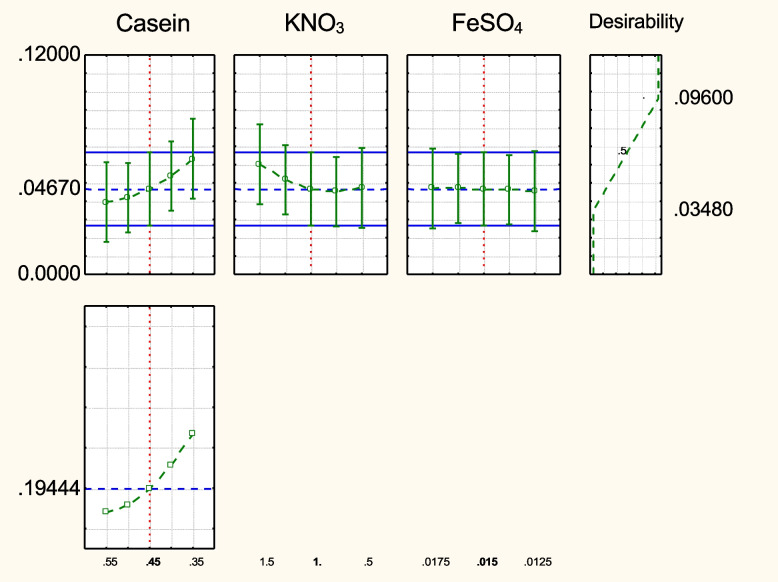
Desirability and prediction profile for the pigment production by *Streptomyces tunisiensis* W4MT573222 in Box-Behnken design

The variance analysis (ANOVA) for the quadratic model response is presented in Table 9S. The tested model was significant at a confidence level equal to 98%. The prediction profile desirability for the production of the pigment is illustrated in Fig. 13 which emphasizes the ranges and tendency of the significant factors that affect pigment production.

**Fig. 14:**
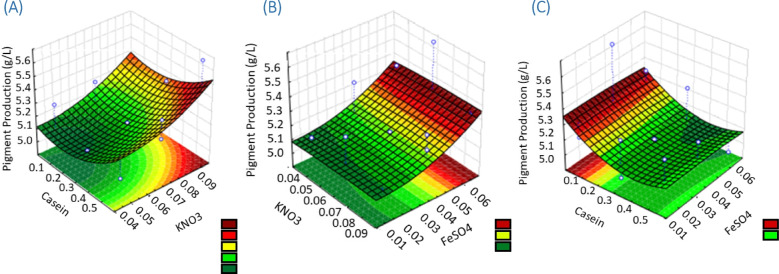
Response surface of the interaction of **A** high pigmentation was recorded for high casein concentration and low KNO_3_ concentration, **B** high casein concentration and slightly high of FeSO_4_ give high pigment production, and **C** low FeSO_4_ and KNO_3_ concentration give high pigment production by *Streptomyces tunisiensis* W4MT57322

The three-dimensional response surface plots are graphical representations of the regression equation, and it shows the interactive effects of variables on pigment production. Higher responses were observed for high concentration of casein and low concentration of KNO_3_ (Fig. 14A), high concentration of casein and slightly high concentration of FeSO_4_ (Fig. 14B), and low concentration of FeSO_4_ and low concentration of KNO_3_ (Fig. 14C).

### Verification of the optimized results

Based on BB and PB experimental results, the pigment production optimum response was predicted with the following composition of the medium (g/L): starch, 5 g; KNO3, 0.88 g; K2HPO_4_, 1 g; MgSO_4_, 0.025 g; FeSO_4_, 0.015 g; casein, 0.65 g; and CaCO_3_, 0.03 g; the medium was adjusted at pH 7, and the flask was incubated at 37 °C on a rotary shaker at 120 rpm. The experiment of confirmation for maximal production of the pigment was applied using the predicted conditions of optimization, where pigment production was calculated after 7 days of incubation at 37 °C. The culture medium of the growth was used as a control. Under the conditions of optimization, the pigment production was 0.12 g/L. These results illustrate that the optimized conditions increased the production of the pigment, and the produced pigment yield was about 12.2-fold accelerated than that noted with the basal culture condition. Matching the predicted pigment production (0.1 g/L) and the observed one (0.12 g/L) under optimal conditions also demonstrated the validity and accuracy of the model in Table 4.

**Table 4:** Verification experiment for pigment production on basal versus optimized medium

**Medium**	**Pigment production (g/L)**	**Fold increase**
Basal medium	0.0098	0
Pre-optimized	0.0467	4.8
Optimized medium	0.12	12.2

### The pigment recovery

The cultivation of* Streptomyces tunisiensis* W4MT573222 was carried out as described by Ibrahim et al. ^1^. The Pigment was produced, extracted, and identified according to the above mentioned methods and then dried to be ready for the subsequent experiments for the applications. It is worth noting that the chemical structure of the partially purified produced pigments of *Streptomyces tunisiensis* W4MT573222 was previously identified by spectroscopy (Raman, FT-IR, EDX) and spectrometry (GC-mass) and previously mentioned by Ibrahim et al. [6].

### In vitro inhibition of albumin denaturation assay

Different concentrations of the extracted pigments were tested for its ability to produce anti-inflammatory agent(s) using a preliminary screening test “inhibition of albumin denaturation assay”. The different concentrations of the partially purified pigments (10, 20, 30, 40, 50, 60, 70, 80, 90, and 1000 µg/ml) were prepared and then tested for their anti-inflammatory activity (the concentrations were prepared according to the safe dose of the pigment on normal human cells). The results presented in Table 5 showed that by increasing the concentration of the pigment which is off the range of the safe dose increases the ability to protect the tested protein (bovine serum albumin) from denaturation compared to diclofenac sodium (a standard anti-inflammatory drug) and represented 40% as shown in Table 5.

**Table 5:** The screening process for anti-inflammatory activity of the test pigments using inhibition of albumin denaturation assay

**The test compound**	**Concentration µg/ml**	**Absorption at 550 nm Wavelength**	**Inhibition of protein denaturation (%)**
**Pigment extract solution**	10	0.991	ND
	20	0.980	ND
	30	0.898	ND
	40	0.851	ND
	50	0.801	ND
	60	0.786	4.1 ± 13
	70	0.733	10.6 ± 15
	80	0.621	24.2 ± 12
	90	0.530	35.36 ± 11
	100	0.490	40 ± 12
	200	0.115	85.9 ± 13
**Diclofenac sodium**	200	0.075	90 ± 9

Bovine serum albumin without any treatment was used as control

## Discussion

Nowadays, there are increasing demands for natural products to replace synthetic products. Pigments are one of these natural products which can be used instead of synthetic colors [21]. Synthetic and natural pigments have been widely used in many fields such as textile industries, agricultural aspects, food production, paper production, cosmetics, and water technology [22]. Natural pigments have different activities like anticancer, antiviral, antibacterial, antifungal, anti-inflammatory, and antioxidant agents [6, 23, 24]. Natural pigments are produced from different sources. Among them, actinomycetes are the most potent natural pigment producers among other microorganisms. Many attempts were used to isolate pigments producing actinomycetes [25]. Actinomycetes are a group of bacteria characterized by unique features. So the main purpose of this work was to isolate pigment-producing actinomycetes with biological activities and also to study the biodiversity of actinomycetes in Abu-Qir Bay, Mediterranean Sea, Egypt [6]. The Abu-Qir Bay, the site of study, receives agricultural drainage water from Edku Lake through the narrow El-Maadya channel (about 200 m long and 2 m deep), and the average of annual discharged water is about 1000 × 10^6^ m^3^/day. Also, freshwater discharged to Abu-Qir Bay through Rosetta Lake is about 1.2 × 10^6^ m^3^/day. The presence of actinomycetes is mainly correlated to agricultural water drainage. So, this bay region is rich with microflora, especially actinomycetes, and was selected for our study. The results indicated that the average counts ranged from 23 CFU/g in Abu-Qir5 to 63CFU/g in Abu-Qir 8, whereas in a previous study by Ghanem et al. who studied the factors influencing the marine actinomycetes distribution in this region, the count was 592 CFU/g [26]. This sharp decrease in the actinomycetes count may be due to the impact of pollution in this region. In previous studies, the counts of the actinomycetes from sediment samples were much higher compared with those recorded in seawater. This might be because the sediment-water interface may react either as a sink or as a potential source of nutrients [26]. So, in this work, sediment samples only were collected; also, the highest counts of actinomycetes in water and sediment samples were detected in dry seasons (autumn and summer); they significantly decreased in spring and disappeared in winter indicating that temperature is most probably the most important environmental factor affecting population sizes. It should also be emphasized that actinomycetes form resistant spores that enable them to survive in dry conditions so that the samples were collected during summer [26]. The used media were starch nitrate medium, starch casein medium, glycerol glycine, and glycerol nitrate media. There was a difference in the number of actinomycetes isolated using the four media. The highest average counts were detected on starch casein medium (52 CFU/g), and the lowest counts were detected on glycerol glycine (25 CFU/g). Ghanem et al.^12^ used starch nitrate, starch casein, glycerol glycine, and chitin when quantifying the actinomycete population in Abu-Qir sediment. They concluded that none of the four tested media was ideal, whereas the most appropriate were starch nitrate, starch-casein, and glycerol glycine giving the highest recorded numbers. Starch nitrate was the most suitable medium which gave high counts. Moreover, it supported the isolation of different types of actinomycetes as judged by different morphological characters and pigmentation. Padma et al. [27] mentioned that 15 different isolates were obtained from 2 different soil samples, 10 isolates from terrestrial soil, and 5 isolates from marine soil samples. They showed better and more efficient growth in starch casein agar medium (SCA). The actinomycete AR-ITM02 was well grown on starch casino agar medium at 30 °C. In this study, out of more than 60 isolates, 12 different isolates were chosen arbitrarily representing all colony morphologies observed from each sample isolated on SCA [28]. All of them were able to grow in inorganic salts starch agar medium and starch nitrate medium, and most of them utilized yeast malt extract agar, Czapek Dox Agar, Krassilnikov agar, and oatmeal agar. The optimum pH range was 7–9, and the temperature range was 25–40 °C. All sugars such as starch, lactose, mannitol, maltose, glucose, and fructose were utilized. The utilization of different sugars was an important step in the characterization and identification of actinomycetes. The actinomycete ARITM02 used different types of carbon sources for growth as arabinose, glucose, mannose, xylose, and fructose, while rhamnose, sucrose, and raffinose were not used for the growth [28]. In this study, 50% of the isolates (six isolates out of 13) had diffusible pigments. Two isolates (9 & W2) had violet color, another two had brown color (H & 28), one had green color (W4), and the latter had beige brown (W1). Based on the previous studies, isolate W4 was carefully selected as the most promising pigment and antimicrobial agent producer as Ibrahim et al. [6]. The results obtained in the current work indicated that the pigment showed an anti-inflammatory effect and prevented the denaturation of BSA protein at a concentration much higher than the safe dose and increased with increasing the pigment concentration, which indicates that the pigments interacted with the aliphatic regions around the lysine residue on the BSA. This could be an interesting anti-inflammatory with anticancer activity such as polyphenols, phenyl prostanoids, and disulfides [6]. In addition, Heo et al. [29] suggested the usage of fucoxanthin (xanthophyll), which is one of two major divisions of the carotenoid group, as a useful therapeutic approach for various inflammatory diseases.

## Conclusion

The utilization of marine actinomycetes as a source for novel bioactive compounds received attention in the last few years; obtaining powerful biologically active metabolites such as actinomycetes is challenging and an interesting platform for many scientists. Despite the ability of actinomycetes to grow under unusual conditions, these microorganisms have the ability to produce different industrial compounds, for example, pigments. Different ecological habitats indicated the presence of actinomycetes species, for example, marine environment. So, we were interested in this study with the isolation and characterization of different species of actinomycetes from Abu-Qir Bay, Mediterranean Sea. Egypt selected the most pigment-producing isolate for studying biological activity of the produced pigment.

### Supplementary Information

**Additional file 1: Table 1S.** The characteristic features of cluster A and the percentages of positive results. **Table 2S.** The characteristic features of cluster B and the percentages of positive results. **Table 3S.** The characteristic features of cluster C and the percentages of positive results. **Table 4S.** The characteristic features of cluster D and the percentages of positive results. **Table 5S.** Phenotypic characteristics of the single clusters. **Table 6S.** Experimental variables at different levels used for pigment production using Plackett–Burman design. **Table 7S.** Statistical analysis of Plackett–Burman design results on pigment production. **Table 8S.** Medium components for pigment production and its three levels used in Box-Behenken design. **Table 9S.** Analysis of variance for the fitted quadratic polynomial model.

## Data Availability

All data analyzed during this study are included in this published article.
