# Supplementary material for: Biodiversity and biological applications of marine actinomycetes—Abu-Qir Bay, Mediterranean Sea, Egypt
Source: J Genet Eng Biotechnol. 2023 Nov 28;21:150. doi: 10.1186/s43141-023-00612-8 (PMC10684441; doi:10.1186/s43141-023-00612-8)
Supplement: Supplementary file 1 — Additional file 1: Table 1S. The characteristic features of cluster A and the percentages of positive results. Table 2S. The characteristic features of cluster B and the percentages of positive results. Table 3S. The characteristic features of cluster C and the percentages of positive results. Table 4S. The characteristic features of cluster D and the percentages of positive results. Table 5S. Phenotypic characteristics of the single clusters. Table 6S. Experimental variables at different levels used for pigment production using Plackett–Burman design. Table 7S. Statistical analysis of Plackett–Burman design results on pigment production. Table 8S. Medium components for pigment production and its three levels used in Box-Behenken design. Table 9S. Analysis of variance for the fitted quadratic polynomial model. [file 43141_2023_612_MOESM1_ESM.docx]

**Table 1S: The characteristic features of cluster A and the percentages of positive results.**

|  | **Cluster A** | | | | **Positive results** | |  |
| --- | --- | --- | --- | --- | --- | --- | --- |
| **Characteristic feature** | **Isolate no** |  |  | |  |  |  |
|  | **23** | **15** |  |  | | **%** | |
| **Growth medium** |  |  |  | |  | |  |
| - yeast malt extract agar | - | - |  | | 0 | |  |
| -Inorganic salts starch agar | + | + |  | | 100 | |  |
| -Czapex Dox agar | + | + |  | | 100 | |  |
| -Krassilnikov agar | + | + |  | | 100 | |  |
| -Oat meal agar  - Starch nitrate agar | -  + | +  + |  | | 50  100 | |  |
| **Substrate mycelium** |  |  |  | |  | |  |
| Greyish yellow | - | + |  | | 50 | |  |
| Yellowish white | + | - |  | | 50 | |  |
| Violet | - | - |  | | 0 | |  |
| Green | - | - |  | | 0 | |  |
| Brown | - | - |  | | 0 | |  |
| **Aerial mycelium** |  |  |  | |  | |  |
| Grey | + | - |  | | 50 | |  |
| Yellow | - | + |  | | 50 | |  |
| Cream | - | - |  | | 0 | |  |
| White | - | - |  | | 0 | |  |
| Pale violet | - | - |  | | 0 | |  |
| **Diffusible pigments** | - | - |  | |  | |  |
| **Temperature (**°C**)**  25-30 | + | + |  | | 100 | |  |
| 40 | + | + |  | | 100 | |  |
| 50 | - | + |  | | 50 | |  |
| **Growth pH** |  |  |  | |  | |  |
| 5-6 | - | + |  | | 50 | |  |
| 7-9 | + | + |  | | 100 | |  |
| **Sugar utilization** |  |  |  | |  | |  |
| Starch | + | + |  | | 100 | |  |
| Lactose | + | + |  | | 100 | |  |
| Mannitol | + | + |  | | 100 | |  |
| Maltose | + | + |  | | 100 | |  |
| D-glucose  Fructose | +  + | +  + |  | | 100  100 | |  |
| **Growth in presence of NaCl (%)** |  |  |  | |  | |  |
| 0 | - | + |  | | 50 | |  |
| 4 | + | + |  | | 100 | |  |
| 7 | + | + |  | | 100 | |  |
| 10 | + | + |  | | 100 | |  |
| 13 | - | - |  | | 0 | |  |
| **Biochemical tests** |  |  |  | |  | |  |
| Protease | + | + |  | | 100 | |  |
| Lipase | + | + |  | | 100 | |  |
| Urease | - | - |  | | 0 | |  |
| Catalase | + | + |  | | 100 | |  |
| Chitinase | + | + |  | | 100 | |  |
|  |  |  |  | |  | |  |
| **Hydrolysis of** |  |  |  | |  | |  |
| Cellulose | + | + |  | | 100 | |  |
| Gelatin | - | - |  | | 0 | |  |

**Table 2S: The characteristic features of cluster B and the percentages of positive results.**

| **Cluster B** | | | | |  |
| --- | --- | --- | --- | --- | --- |
| **Characteristic feature** | **Isolate no** | |  | **Positive results** |  |
|  | **50** | **8** |  | **%** |  |
| **Growth medium** |  |  |  |  |  |
| - yeast malt extract agar | + | + |  | 100 |  |
| -Inorganic salts starch agar | + | + |  | 100 |  |
| -Czapex Dox agar | + | + |  | 100 |  |
| -Krassilnikov agar | + | + |  | 100 |  |
| -Oat meal agar  - Starch nitrate agar | +  + | -  + |  | 50  100 |  |
| **Substrate mycelium** |  |  |  |  |  |
| Greyish yellow | + | + |  | 100 |  |
| Yellowish white | - | - |  | 0 |  |
| Violet | - | - |  | 0 |  |
| Green | - | - |  | 0 |  |
| Brown | - | - |  | 0 |  |
| **Aerial mycelium** |  |  |  |  |  |
| Cream | + | + |  | 100 |  |
| Yellow | - | - |  | 0 |  |
| Gery | - | - |  | 0 |  |
| White | - | - |  | 0 |  |
| Pale violet | - | - |  | 0 |  |
| **Diffusible pigments** | - | - |  |  |  |
| **Temperature (**°C**)**  25-30 | + | + |  | 100 |  |
| 40 | + | + |  | 100 |  |
| 50 | + | + |  | 100 |  |
| **Growth pH** |  |  |  |  |  |
| 5-6 | - | + |  | 50 |  |
| 7-9 | + | + |  | 100 |  |
| **Sugar utilization** |  |  |  |  |  |
| Starch | + | + |  | 100 |  |
| Lactose | + | + |  | 100 |  |
| Mannitol | + | + |  | 100 |  |
| Maltose | + | + |  | 100 |  |
| D-glucose  Fructose | +  + | +  + |  | 100  100 |  |
| **Growth in presence of NaCl (%)** |  |  |  |  |  |
| 0 | + | - |  | 50 |  |
| 4 | + | + |  | 100 |  |
| 7 | + | + |  |  |  |
| 10 | - | + |  |  |  |
| 13 | - | - |  |  |  |
| **Biochemical tests** |  |  |  |  |  |
| Protease | + | + |  | 100 |  |
| Lipase | + | + |  | 100 |  |
| Urease | + | + |  | 100 |  |
| Catalase | - | - |  | 0 |  |
|  |  |  |  |  |  |
| Chitinase  **Hydrolysis of** | + | + |  | 100 |  |
| Cellulose | + | + |  | 100 |  |
| Gelatin | - | - |  | 0 |  |

**Table 3S: The characteristic features of cluster C and the percentages of positive results.**

|  | **Cluster C** | | | | **Positive results** | |  |
| --- | --- | --- | --- | --- | --- | --- | --- |
| **Characteristic feature** | **Isolate no** | |  | |  |  |  |
|  | **9** | **W2** |  |  | | **%** | |
| **Growth medium** |  |  |  | |  | |  |
| - yeast t malt extract agar | + | + |  | | 100 | |  |
| -Inorganic salts starch agar | + | + |  | | 100 | |  |
| -Czapex Dox agar | + | + |  | | 100 | |  |
| -Krassilnikov agar | + | + |  | | 100 | |  |
| -Oat meal agar  - Starch nitrate agar | +  + | +  + |  | | 100  100 | |  |
| **Substrate mycelium** |  |  |  | |  | |  |
| Violet | + | + |  | | 100 | |  |
| Greyish yellow | - | - |  | | 0 | |  |
| Yellowish white | - | - |  | | 0 | |  |
| Green | - | - |  | | 0 | |  |
| Brown |  |  |  | | 0 | |  |
|  |  |  |  | |  | |  |
| **Aerial mycelium** |  |  |  | |  | |  |
| White | + | - |  | | 50 | |  |
| Pale violet | - | + |  | | 50 | |  |
| Yellow | - | - |  | | 0 | |  |
| Cream | - | - |  | | 0 | |  |
| Grey | - | - |  | | 0 | |  |
| **Diffusible pigments** |  |  |  | |  | |  |
| Violet | + | + |  | | 100 | |  |
| Green | - | - |  | | 0 | |  |
| Brown | - | - |  | | 0 | |  |
| Beige to brown | - | - |  | | 0 | |  |
| **Temperature (**°C**)**  25-30 | + | + |  | | 100 | |  |
| 40 | + | + |  | | 100 | |  |
| 50 | + | + |  | | 100 | |  |
| **Growth pH** |  |  |  | |  | |  |
| 5-6 | + | + |  | | 100 | |  |
| 7-9 | + | + |  | | 100 | |  |
| **Sugar utilization** |  |  |  | |  | |  |
| Starch | + | + |  | | 100 | |  |
| Lactose | + | + |  | | 100 | |  |
| Mannitol | + | + |  | | 100 | |  |
| Maltose | + | + |  | | 100 | |  |
| D-glucose  fructose | +  + | +  + |  | | 100  100 | |  |
| **Growth in presence of NaCl (%)** |  |  |  | |  | |  |
| 0 | - | + |  | | 50 | |  |
| 4 | + | + |  | | 100 | |  |
| 7 | + | + |  | | 100 | |  |
| 10 | + | - |  | | 50 | |  |
| 13 | - | - |  | | 0 | |  |
| Biochemical tests |  |  |  | |  | |  |
| Protease | + | + |  | | 100 | |  |
| Lipase | + | + |  | | 100 | |  |
| Urease | + | + |  | | 100 | |  |
| Catalase | + | + |  | | 50 | |  |
|  |  |  |  | |  | |  |
| Chitinase  **Hydrolysis of** | + | + |  | | 100 | |  |
| Cellulose | + | + |  | | 100 | |  |
| Gelatin | - | - |  | | 0 | |  |

**Table 4S: The characteristic features of cluster D and the percentages of positive results.**

|  | **Cluster D ( 2 isolates)** | | | | **positive results** | |  |
| --- | --- | --- | --- | --- | --- | --- | --- |
| **Characteristic feature** | **Isolate no** | |  | |  |  |  |
|  | **H** | **28** |  |  | | **%** | |
| **Growth medium** |  |  |  | |  | |  |
| - yeast malt extract agar | + | + |  | | 100 | |  |
| -Inorganic salts starch agar | + | + |  | | 100 | |  |
| -Czapex Dox agar | + | - |  | | 50 | |  |
| -Krassilnikov agar | + | + |  | | 100 | |  |
| -Oat meal agar  - Starch nitrate agar | +  + | +  + |  | | 100  100 | |  |
| **Substrate mycelium** |  |  |  | |  | |  |
| Brown | + | + |  | | 100 | |  |
| Greyish yellow | - | - |  | | 0 | |  |
| Yellowish white | - | - |  | | 0 | |  |
| Green | - | - |  | | 0 | |  |
| Violet | - | - |  | | 0 | |  |
| **Aerial mycelium** |  |  |  | |  | |  |
| White | + | + |  | | 100 | |  |
| Cream | - | - |  | | 0 | |  |
| Grey | - | - |  | | 0 | |  |
| Pale Violet | - | - |  | | 0 | |  |
| Yellow | - | - |  | | 0 | |  |
| **Diffusible pigments** |  |  |  | |  | |  |
| Brown | + | + |  | | 100 | |  |
| Green | - | - |  | | 0 | |  |
| Violet | - | - |  | | 0 | |  |
| Beige to Violet | - | - |  | | 0 | |  |
| **Temperature (**°C**)**  25-30 | + | + |  | | 100 | |  |
| 40 | + | + |  | | 100 | |  |
| 50 | - | - |  | | 0 | |  |
| **Growth pH** |  |  |  | |  | |  |
| 5-6 | + | + |  | | 100 | |  |
| 7-9 | + | + |  | | 100 | |  |
| **Sugar utilization** |  |  |  | |  | |  |
| Starch | + | + |  | | 100 | |  |
| Lactose | + | + |  | | 100 | |  |
| Mannitol | + | + |  | | 100 | |  |
| Maltose | + | + |  | | 100 | |  |
| D-glucose  Fructose | +  + | +  + |  | | 100  100 | |  |
| **Growth in presence of NaCl (%)** |  |  |  | |  | |  |
| 0 | + | - |  | | 50 | |  |
| 4 | + | + |  | | 100 | |  |
| 7 | + | + |  | | 100 | |  |
| 10 | + | - |  | | 50 | |  |
| 13 | - | - |  | | 0 | |  |
| **Biochemical tests** |  |  |  | |  | |  |
| Protease | + | + |  | | 100 | |  |
| Lipase | + | + |  | | 100 | |  |
| Urease | - | + |  | | 50 | |  |
| Catalase | - | - |  | | 0 | |  |
| Chitinase | + | + |  | | 100 | |  |
|  |  |  |  | |  | |  |
| **Hydrolysis of** |  |  |  | |  | |  |
| Cellulose | + | + |  | | 100 | |  |
| Gelatin | - | - |  | | 0 | |  |

**Table 5S: Phenotypic characteristics of the single clusters.**

| **Characteristic feature** | **Single clusters** | | | | | | | | | | | |  |
| --- | --- | --- | --- | --- | --- | --- | --- | --- | --- | --- | --- | --- | --- |
|  |  |  |  |  | **Isolate no** | | | | | |  |  |  |
|  |  |  |  | **38** |  | **W1** | |  | **W3** |  | **W4** |  |  |
| **Growth medium** |  |  |  |  |  |  |  | |  |  |  |  |  |
| Yeast malt extract agar |  |  |  | + |  | + |  | | - |  | + |  |  |
| Inorganic salts starch agar |  |  |  | + |  | + |  | | + |  | + |  |  |
| Cazpex Dox agar |  |  |  | + |  | + |  | | - |  | + |  |  |
| Krassilinkov agar |  |  |  | + |  | + |  | | - |  | - |  |  |
| Oat meal agar |  |  |  | + |  | + |  | | + |  | + |  |  |
| Starch nitrate agar |  |  |  | + |  | + |  | | + |  | + |  |  |
| **Substrate mycelium** |  |  |  |  |  |  |  | |  |  |  |  |  |
| Yellowish white |  |  |  | + |  | - |  | | - |  | - |  |  |
| Cream |  |  |  | - |  | - |  | | + |  | - |  |  |
| Green |  |  |  | - |  | - |  | | - |  | + |  |  |
| Brown |  |  |  | - |  | + |  | | - |  | - |  |  |
| Greyish yellow  **Aerial mycelium** |  |  |  | - |  | - |  | | - |  | - |  |  |
| White |  |  |  | - |  | + |  | | + |  | - |  |  |
| Yellow |  |  |  | + |  | - |  | | - |  | + |  |  |
| Cream |  |  |  |  |  |  |  | |  |  |  |  |  |
| Grey |  |  |  | - |  | - |  | | - |  | - |  |  |
| Pale yellow |  |  |  | - |  | - |  | | - |  | - |  |  |
| **Diffusible pigments** |  |  |  |  |  |  |  | |  |  |  |  |  |
| Beige to brown |  |  |  | - |  | + |  | | - |  | - |  |  |
| Green |  |  |  | - |  | - |  | | - |  | + |  |  |
| Violet |  |  |  | - |  | - |  | | - |  | - |  |  |
| Brown |  |  |  | - |  | - |  | | - |  | - |  |  |
| **Temperature (**°C**)** |  |  |  |  |  |  |  | |  |  |  |  |  |
| 25-30 |  |  |  | + |  | + |  | | + |  | + |  |  |
| 40 |  |  |  | + |  | + |  | | + |  | + |  |  |
| 50 |  |  |  | - |  | + |  | | + |  | + |  |  |
| **Growth pH** |  |  |  |  |  |  |  | |  |  |  |  |  |
| 5-6 |  |  |  | + |  | + |  | | + |  | + |  |  |
| 7-9 |  |  |  | + |  | + |  | | + |  | + |  |  |
| **Sugar utilization** |  |  |  |  |  |  |  | |  |  |  |  |  |
| Starch |  |  |  | + |  | + |  | | + |  | + |  |  |
| Lactose |  |  |  | + |  | + |  | | + |  | + |  |  |
| Mannitol |  |  |  | + |  | + |  | | + |  | + |  |  |
| Maltose |  |  |  | + |  | + |  | | + |  | + |  |  |
| D-glucose |  |  |  | + |  | + |  | | + |  | + |  |  |
| Fructose |  |  |  | + |  | + |  | | + |  | + |  |  |
| **Growth in presence of NaCl (%)** |  |  |  |  |  |  |  | |  |  |  |  |  |
| 0 |  |  |  | + |  | + |  | | + |  | + |  |  |
| 4 |  |  |  | + |  | + |  | | + |  | + |  |  |
| 7 |  |  |  | + |  | + |  | | + |  | + |  |  |
| 10 |  |  |  | + |  | + |  | | - |  | - |  |  |
| 13 |  |  |  | - |  | - |  | | - |  | - |  |  |
| **Biochemical tests** |  |  |  |  |  |  |  | |  |  |  |  |  |
| Protease |  |  |  | + |  | + |  | | + |  | + |  |  |
| Lipase |  |  |  | + |  | + |  | | + |  | + |  |  |
| Urease |  |  |  | + |  | - |  | | - |  | + |  |  |
| Catalase |  |  |  | - |  | + |  | | - |  | + |  |  |
| Chitinase |  |  |  | + |  | + |  | | + |  | + |  |  |
| **Hydrolysis of** |  |  |  |  |  |  |  | |  |  |  |  |  |
| Cellulose |  |  |  | + |  | + |  | | + |  | + |  |  |
| Gelatin |  |  |  | - |  | + |  | | + |  | + |  |  |
|  |  |  |  |  |  |  |  | |  |  |  |  |  |

**Table 6S: Experimental variables at different levels used for pigment production using Plackett–Burman design.**

|  | Levels (g/L) |  |  |
| --- | --- | --- | --- |
| Factor | **-1** | **0** | **+1** |
| Starch | 5 | 10 | 20 |
| KNO_3_ | 1 | 2 | 3 |
| K_2_HPO_4_ | 1 | 2 | 3 |
| MgSO_4_.7H_2_O | 0.025 | 0.05 | 0.075 |
| FeSO_4_ | 0.005 | 0.01 | 0.015 |
| Casein | 0.15 | 0.3 | 0.45 |
| CaCO_3_ | 0.01 | 0.02 | 0.03 |

**Table 7S: Statistical analysis of Plackett–Burman design results on pigment production.**

|  | Main  effect | Coefficient  s | Standard  Error | *T*- Stat | *P*-value | Significanc  e % |
| --- | --- | --- | --- | --- | --- | --- |
| Intercept |  | 0.010978 | 0.000416 | 26.36308 | 0.02413  7 |  |
| Starch | -0.0005 | -0.00025 | 0.000442 | -0.56604 | 0.67209  4 | 32.8 |
| K NO3 | -0.006 | -0.003 | 0.000442 | -6.79245 | 0.09305  6 | 90.7 |
| K2HPO4 | -0.00565 | -0.00283 | 0.000442 | -6.39623 | 0.09873  1 | 90.1 |
| MgSO4 | -0.00205 | -0.00103 | 0.000442 | -2.32075 | 0.25901 | 74.1 |
| FeSO4 | 0.006 | 0.003 | 0.000442 | 6.792453 | 0.09305  6 | 90.7 |
| Casein | 0.0104 | 0.0052 | 0.000442 | 11.77358 | 0.05394  2 | 94.6 |
| CaCO3 | 0.00055 | 0.000275 | 0.000442 | 0.622642 | 0.64546  5 | 35.5 |

**Table 8S: Medium components for pigment production and its three levels used in Box-Behenken design.**

| Variables(g/L) | Experimental values | |  |
| --- | --- | --- | --- |
|  | -1 | 0 | +1 |
| Casein (X_1_) | 0.35 | 0.45 | 0.55 |
| KNO_3_(X_2_) | 0.5 | 1 | 1.5 |
| FeSO_4_(X_3_) | 0.0125 | 0.015 | 0.0175 |

**Table 9S: Analysis of variance for the fitted quadratic polynomial model.**

|  | Coefficients | Standard Error | *t* Stat | *P*-value |
| --- | --- | --- | --- | --- |
| Intercept | -0.16 | 0.10 | -1.68 | 0.15 |
| X1 | 0.04 | 0.20 | 0.20 | 0.85 |
| X2 | 0.22 | 0.03 | 7.09 | 0.00 |
| X3 | 9.83 | 9.62 | 1.02 | 0.35 |
| X1X2 | -0.36 | 0.04 | -10.15 | 0.00 |
| X1X3 | 1.20 | 7.15 | 0.17 | 0.87 |
| X2X3 | -8.28 | 1.43 | -5.79 | 0.00 |
| 2  X_1_ | 0.47 | 0.19 | 2.53 | 0.05 |
| 2  X_2_ | 0.03 | 0.01 | 3.79 | 0.01 |
| 2  X_3_ | -80.00 | 297.77 | -0.27 | 0.80 |
